# Lumbar Puncture Alleviates Chorea in a Patient with Huntington’s Disease and Normal Pressure Hydrocephalus

**DOI:** 10.3233/BEN-2009-0239

**Published:** 2009-12-23

**Authors:** Peyman Shirani, Alicia R. Salamone, Elham Lahijani, Michele K. York, Paul E. Schulz

**Affiliations:** ^1^Department of NeurologyBaylor College of MedicineHoustonTXUSA; ^2^The Methodist HospitalHoustonTXUSA

**Keywords:** Chorea, Huntington's disease, hydrocephalus

## Abstract

A 44-year-old African-American male was admitted to our hospital after a suicide attempt. He had depression, poor cognitive function, choreiform movements, difficulty pronouncing words, and difficulty walking. His symptoms had worsened markedly over several months. Chorea lead to genetic testing that confirmed a diagnosis of Huntington Disease (HD). A CT scan of the head showed wider ventricles than is typical of HD. The head CT and gait change suggested normal pressure hydrocephalus (NPH). Lumbar puncture (LP) led to improved neuropsychologic test scores and walking thereby supporting the diagnosis of NPH. Surprisingly, the LP also led to an 80% improvement of chorea. There are two other reports of an association between HD and NPH. NPH should be considered in HD patients with atypical symptoms, such as the inability to walk or rapid progression, as its treatment may lead to improved cognition, gait, and chorea.

## 1. Introduction

Huntington's disease (HD) is an inherited, progressive neurodegenerative disorder characterized by chorea, involuntary movements, personality changes and dementia. This disorder commonly occurs in mid-life and leads to death within 10–15 years [1]. HD is caused by a trinucleotide repeat expansion in the gene coding for Huntingtin protein (Htt), and patients become symptomatic for the disease when this repeat expands more than 40 times [2]. Current treatments for this disorder are palliative [3].

Normal pressure hydrocephalus (NPH) results from impaired absorption of the cerebrospinal fluid, which can be idiopathic or result from a number of underlying causes. The diagnosis of NPH is based on clinical and radiographic findings. It often presents with the classic triad of cognitive deterioration, abnormal gait and urinary incontinence. NPH may respond to ventricular shunting with improvements in gait more than cognitive impairment and urinary dysfunction [5]. Choreic movement, while commonly seen in advanced HD, is rarely noted in NPH; however, chorea can occur if the caudate nucleus is compromised [4].

We report a case of HD with NPH. Two cases published nearly thirty years ago reported alleviation of choreic movement with lumbar puncture (LP) in HD [6, 7]. Therefore, our patient underwent a LP. It improved both his symptoms of NPH and his chorea.

## 2. Case report

A 44-year old African-American male was admitted to the hospital following a suicide attempt. During the preceding four years, the patient reported memory

ISSN 0953-4180/09/$17.00 © 2009 – IOS Press and the authors. All rights reserved

Table 1
Neuropsychological evaluation pre and post lumbar puncture

| | Pre-LP | Post-LP | 95% RCI criterion | Difference Pre/Post LP |
|---|---|---|---|---|
| **Mental Status** | | | | |
| MMSE (0–30) | 18 | 23 | 1.30 | 5* |
| **Visuospatial** | | | | |
| Clock Drawing (0–10) | 3 | 5 | 0.80 | 2* |
| **Attention** | | | | |
| TSAT time (0–480 sec) | 480 | 310 | 18.9 | 170* |
| TSAT errors (0–45) | 45 | 18 | 0.82 | 27* |
| Digit Span (0–30) | 0 | 12 | 1.90 | 12* |
| **Language** | | | | |
| Verbal Fluency (no max) | 1 | 11 | 12.0 | 10 |
| Semantic Fluency (no max) | 4 | 12 | 4.10 | 8* |
| Boston Naming (0–60) | 27 | 48 | 2.70 | 21* |
| **Verbal Memory** | | | | |
| HVLT Learning (0–36) | 0 | 11 | 0.74 | 11* |
| Delayed Recall (0–12) | 0 | 1 | 0.66 | 1* |
| Recognition Discrim (0–12) | 0 | 0 | 2.67 | 0 |

Lumbar Puncture (LP); Mini-Mental Status Exam (MMSE); WAIS-III Digit Span (Digit Span); Test of Sustained Attention (TSAT); Verbal Fluency (FAS); Semantic Fluency-Animals; Boston Naming Test (BNT); Hopkins Verbal Learning Test-Revised (HVLT-R).
*Significantly reliable difference between pre and post lumbar puncture.

loss, depression and a seizure. Six months prior to admission, he developed abnormal movements of the limbs. A few weeks prior to admission, he developed gait problems that required a wheel chair, and he started to have urinary incontinence.

Past medical history was negative for head trauma, meningitis and diabetes. The patient denied tobacco, alcohol and recreational drug usage. He was married and lived with his wife. Medications included phenytoin, which was started four years prior due to the occurrence of an unknown type of seizures.

The patient's neurological examination demonstrated involuntary movements of the face, tongue, head, trunk and bilateral upper extremities that were sudden and choreiform. The patient had a magnetic gait, difficulty arising from a chair, and an increase in falls over the past few weeks. Significant dysarthria was noted on exam and he showed almost constant guttural vocalizations. At times, his speech was incomprehensible.

The patient's neuropsychological evaluation revealed global cognitive impairment with deficits in mental status, visuospatial abilities, verbal memory, verbal and semantic fluency, and attention and concentration (Table, Pre-LP). The patient reported that he was unable to perform any of the tasks on first presentation, but with prompting from the examiner, he was able to complete some of the tasks. Due to the patient's severe vocalizations, he was unable to perform two of the measures administered: Digit Span and Test of Sustained Attention. Due to choreiform movements, he was unable to hold a pencil and draw.

The patient's background and symptoms raised the suspicion of HD. He revealed that involuntary movements occurred in both his mother and grandmother beginning at a similar age. Genetic testing revealed an expansion of 48 repeats in the gene encoding Htt, thereby confirming HD. All other blood tests were normal, including the phenytoin level. The patient was started on duloxetine hydrochloride for depression.

A head CT scan showed enlarged ventricles and diffuse atrophy suggesting NPH and HD. To test for NPH, 50 cc of cerebrospinal fluid was removed by LP. Cerebrospinal pressure was normal (CSF pressure 100 mm water). There were improvements in classic NPH signs, including gait. Surprisingly, the LP also produced improvement in his involuntary movements, vocalizations and dysarthria.

His movement disorder was rated by the Unified Huntington's Disease Rating Scale (UDHRS) motor score, which was administrated before and the day after the LP. His UHDRS motor subsection score improved by 80 percent. His vocalizations were reduced following LP and his speech was noticeably more intelligible.

The patient underwent repeat neuropsychological testing the day after the LP. The patient demonstrated improvements in mental orientation, verbal memory, attention, and confrontation naming in a pattern typical following treatment for NPH. The improved scores were further examined using the reliable changes in-

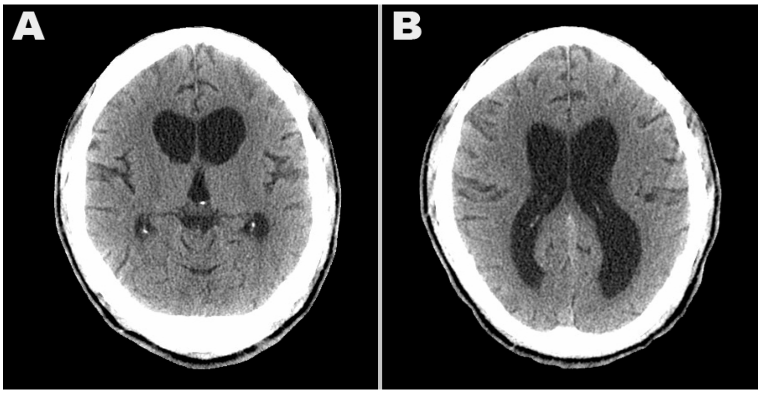

Fig. 1. (A). The CT scan demonstrated caudate atrophy, suggestive of Huntington's disease. (B) Enlarged lateral ventricles were consistent with a secondary diagnosis of NPH.

dexes (RCIs) [8], a statistical method that adjusts for practice effects and measures the degree to which a test score changes between two time periods beyond the range that could be attributed to measurement variability of the neuropsychological test itself. A 95% confidence interval adjusted for neuropsychological practice effects was calculated revealing reliable improvements in the previously mentioned cognitive domains (Table, Post-LP). While improved from his baseline level, his scores continued to fall within the impaired range when compared to normative data for his age and education. The only exception was Digit Span, which was low average for his age.

## 3. Discussion

We report an unusual case of HD with NPH. Following LP, the patient's choreiform movements and vocalizations improved, as did the classic symptoms associated with NPH, such as cognitive impairment and gait.

We have two hypotheses to account for the improvement in involuntary movements after LP. One is that there might have been a change in CSF effects on the caudate nucleus, already damaged by HD. Alternately, there may have been alleviation of chorea caused by NPH, which rarely produces chorea itself. Nearly thirty years ago, there were two reports of the alleviation of chorea following LP in patients with NPH and HD [6, 7]. To our knowledge, LPs have not been investigated as a potential treatment for chorea outside of the setting of NPH.

In the future, it may be useful to investigate whether LPs and ventriculoperitoneal shunt placement are helpful for the debilitating choreiform movements of HD outside of patients with concomitant NPH. Additionally, brain imaging in patients with HD might be inspected for NPH, which might be aggravating chorea. Imaging may be particularly helpful when unusual symptoms develop or when HD patients experience a rapid decline in gait or cognition.

## References

[1] E.J. Wild and S.J. Tabrizi. The differential diagnosis of chorea, *Pract Neurol* **7** (2007), 360–373.

[2] D.C. Rubinsztein, J. Leggo, R. Coles, L.E. DeLisi, C. Walsh, S. Jain and E.S. Paykel, Phenotypic characterization of individuals with 30–40 CAG repeats in the Huntington disease (HD) gene reveals HD cases with 36 repeats and apparently normal elderly individuals with 36–39 repeats, *Am J Hum Genet* **59** (1996), 16–22.

[3] R.M. Bonelli, G.K. Wenning and H.P. Kapfhammer, Huntington's disease: present treatments and future therapeutic modalities, *Int Clin Psychopharmacol* **19** (2005), 51–62.

[4] N.C. Voermans, P.J. Schutte and B.R. Bloem, Hydrocephalus induced chorea, *J Neurol Neurosurg Psychiatry* **78** (2007), 1284–1285.

[5] G.L. Gallia, D. Rigamonti and M.A. Williams, The diagnosis and treatment of idiopathic normal pressure hydrocephalus, *Nat Clin Pract Neurol* **2** (2006), 375–381.

[6] B.H. Tang, A. Lieberman and R. Rovit, Huntington's chorea associated with normal pressure hydrocephalus, *Eur Neurol* **19** (1975), 189–194.

[7] A. Jayaraman, M. Garofalo, Jr., H. Donnenfeld and J.G. Chusid, Huntington's chorea and normal-pressure hydrocephalus, *N Y State J Med* **78** (1978) 1465–1468.

[8] N.S. Jacobson and P. Turax, significance: A statistical approach to defining meaningful change in psychotherapy research, *J Consulting Clin Psych* **59** (1991), 12–19.
